# Can prolyl endopeptidase reduce the IgE-reactivity of gluten proteins?

**DOI:** 10.1186/2045-7022-5-S3-P117

**Published:** 2015-03-30

**Authors:** Frances Smith, Peter Shewry, Neil Carr, Joan Tomas, Phil Padfield, Clare Mills

**Affiliations:** 1 The University of Manchester, Manchester, United Kingdom; 2 Rothamsted Research, Harpenden, United Kingdom; 3 DSM Biotechnology Centre, Heerlen, The Netherlands; 4 Department of Pneumology and Respiratory Allergy, Barcelona, Spain

## Background

Coeliac disease and IgE-mediated allergy to wheat are immune-mediated conditions which are thought to be triggered by digestion-resistant wheat proteins. Recently, a prolyl endopeptidase from *Aspergillus niger* (AnPEP) has been identified as being able to accelerate breakdown of gluten in food using an *in vitro* digestion system. Such digests have been analysed in terms of coeliac disease t-cell epitope levels[1] but have not yet been considered regarding the impact on IgE-reactivity. The amount of AnPEP required to be effective also has yet to be investigated.

## Methods

We have used an *in vitro* batch gastric digestion model to break down a bread matrix with different amounts of AnPEP. SDS PAGE and immunoblots have been used to monitor protein digestion, and mass spectrometry has allowed for epitopes important to IgE-mediated wheat allergy and coeliac disease to be tracked. Sera were obtained from a Spanish patient panel with allergy to wheat induced by exercise and/or NSAIDs and used for additional immunoblots.

## Results

SDS PAGE and immunoblotting with anti-gluten antibodies show more effective breakdown of gliadins and LMW glutenins in the presence of AnPEP. The impact of digestion on the IgE-reactivity of gluten by immunoblotting has been correlated with resistant proteins and peptides mapped by mass spectrometry. Use of 200 mg AnPEP/ g substrate showed the largest difference in digestion kinetics of gluten proteins however increased breakdown was still visible using 20 mg AnPEP/ g substrate.

## Conclusions

AnPEP alters the patterns and pathways of gluten in a simulated gastroduodenal digestion system. The potential application of such enzymes to modify the allergenic potential of cereals is discussed.
